# In silico study, synthesis, and antineoplastic evaluation of thiazole-based sulfonamide derivatives and their silver complexes with expected carbonic anhydrase inhibitory activity

**DOI:** 10.25122/jml-2023-0180

**Published:** 2023-12

**Authors:** Esraa Mahdi Naji, Noor Hatef Naser, Sahar Aqeel Hussein

**Affiliations:** 1 Pharmaceutical Chemistry Department, Faculty of Pharmacy, Kufa University, Najaf, Iraq; 2 Pharmaceutical Chemistry Department, College of Pharmacy, Al-Zahraa University for Women, Karbala, Iraq

**Keywords:** carbonic anhydrase, sulfonamide, molecular docking

## Abstract

This study aimed to design, synthesize, and evaluate the cytotoxic activity of novel thiazole-sulfanilamide derivatives, specifically compounds M3, M4, and M5, through molecular docking and biological assays. The synthesis utilized essential chemical compounds, including sulfanilamide, chloro-acetyl chloride, thiourea, derivatives of benzaldehyde, and silver nitrate. The docking study was carried out using Molecular Operating Environment (MOE) software, and cytotoxic activity was predicted by MTT assay. The synthesized compounds demonstrated a reduction in the viability of cancer cells. Compound M5 had an IC50 of 18.53 µg/ml against MCF-7 cells, comparable to the IC50 of cisplatin. Additionally, compounds M3 and M4 had higher S scores than acetazolamide, indicating greater binding affinity to the active pocket of the receptor. Incorporating the thiazole ring in the synthesized compound augmented their flexibility and affinity for binding to the receptor. The inclusion of the metal complex additionally heightened the compounds’ capacity to impede cellular growth.

## INTRODUCTION

Cancer stands as a significant global challenge, responsible for a substantial number of deaths and hindering efforts to improve life expectancy worldwide. According to the World Health Organization's (WHO) 2019 data, cancer ranks among the top two causes of death before the age of 70 in 112 out of 183 countries. Additionally, it holds the position of the third or fourth leading cause of death in 23 other countries [1]. With respect to breast cancer, specific subtypes have become resistant to medications over time despite the availability of various therapeutic options. Such resistance can lead to treatment ineffectiveness and disease advancement [2]. Overcoming this obstacle remains a significant challenge that requires further investigation and innovative solutions. One of the new targets for tumor cells, aimed at addressing the tumor microenvironment to mitigate chemotherapy resistance, is the group of enzymes known as carbonic anhydrases (CAs). CAs are a widespread group of metalloenzymes found across various species and are encoded by eight gene families, from α- to ι [3, 4]. These enzymes facilitate the reversible conversion of carbon dioxide into a bicarbonate ion and a proton, a vital reaction involved in numerous physiological functions [5]. There are fifteen α-carbonic anhydrase isoforms (hCAs) that exhibit variations in catalytic efficacy, subcellular and tissue distribution, and human physiological function. Of these, twelve isoforms possess catalytic activity (hCA I, II, III, IV, VA, VB, VI, VII, IX, XII, XIII, XIV) [6]. In response to hypoxia-inducible factor-1 (HIF-1), the expression of certain CAs, particularly isoform IX, which is almost undetectable in normal cells, along with isoform XII, increases under hypoxic conditions in tumors. These isoforms have an essential function in conjunction with anaerobic glycolysis, contributing to the acidification of the tumor microenvironment thereby influencing tumor survival and proliferation [7]. Therefore, targeting this enzyme has emerged as a promising area of investigation in chemotherapy [8]. Given their selectivity for cancer cells, these enzymes present specific therapeutic targets that could minimize adverse effects [9]. The zinc-binding group represents a traditional form of inhibitor for this enzyme. Carbonic anhydrase inhibitors (CAIs) coordinate with the essential Zn^+2^ within the active site of the enzyme [10, 11]. Zn^+2^ may adopt tetrahedral or trigonal bipyramidal geometries in the inhibitory process. Sulfonamides are recognized examples of CAIs that utilize this binding mode [12, 13]. Gaining insights into the structural intricacies of the active site of carbonic anhydrase offers a promising pathway for designing specific inhibitors. Among the diverse range of carbonic anhydrase inhibitors, aryl-sulfonamides emerge as a promising category, known for their strong binding affinity with CAs. While these compounds can be synthesized relatively easily, achieving a high level of selectivity remains a significant challenge. Nonetheless, the utilization of the "tail approach," involving the modification of a substituent on the benzene ring carrying the sulfonamide group, has demonstrated the potential to achieve significant selectivity towards the desired target CAs [14]. Similarly, thiazole is a distinct ring structure containing nitrogen and sulfur atoms within five-membered heteroaryl ring systems. This characteristic makes thiazole highly versatile in a wide range of biochemical actions and chemical reactions. The existence of an H-proton at C-2 on the thiazole ring augments its reactivity, rendering it a crucial synthon for synthesizing a diverse array of chemical compounds [15, 16]. In the field of cancer drugs, several compounds containing a thiazole ring are used in in vitro and in vivo studies. Examples include Bleomycin, Tiazofurin, and Dabrafenib, with alpelisib (Pigray^®^) receiving FDA approval in 2019 to treat specific types of breast cancer [16].

Furthermore, metal complexes derived from sulfonamide carbonic anhydrase inhibitors display a remarkable ability to exhibit inhibitory potency 10 to 100 times greater than the original sulfonamide compound. This heightened inhibition is attributed to a dual-action mechanism involving sulfonamide anions and metal ions. Dissociation of the coordination compounds achieves the desired effect at low concentrations. This approach involves the creation of sulfonamide anions, which attach themselves to the Zn (II) ion at the active site of the enzyme. In parallel, including metal ions impedes the proton shuttle residues of the carbonic anhydrase [17]. This study aimed to design and synthesize novel compounds integrating sulfonamide as a CAI and a thiazole ring. Furthermore, we aimed to synthesize a silver complex using these compounds and evaluate their cytotoxic activity using computational analysis.

## MATERIAL AND METHODS

The chemicals and anhydrous solvents used in this study were sourced from various suppliers, including Merck, Reidal Dehean, Sigma-Aldrich, and Thomas Baker. The Thomas-Hoover apparatus was utilized for detecting melting points through the capillary tube technique. Ascending thin-layer chromatography was employed to verify the progression of reactions, purify the synthesized compounds, and calculate the retention factor (Rf) values, with methanol and acetone (1:1) acting as the mobile phase [18]. Shimadzu spectrophotometer was used by the College of Pharmacy, University of Kufa, to scan for FT-IR and estimate spectra using potassium bromide (KBr) discs. Bruker equipment was utilized by the Mashhad University of Medical Sciences to capture proton nuclear magnetic resonance (^1^H-NMR) and carbon-13 nuclear magnetic resonance (^13^C-NMR) data, using DMSO-d6 as the solvent.

### Chemical synthesis

The preparation of 2-Chloro-N-(4-sulfamoyl phenyl) acetamide C_8_H_9_C_l_N_2_O_3_S (M1) was made by reacting sulfanilamide with chloro-acetyl chloride [19]. Sulfanilamide (2gm, 11.6 mmol) was dissolved in a 40 ml mixture of dimethylformamide (DMF) and benzene in a 1:3 ratio, and then 1.6 ml (11.6 mmol) of triethylamine (TEA) was added. The reaction mixture was continuously stirred on an ice bath to maintain a low temperature, and chloro-acetyl chloride (0.92 ml, 11.6 mmol in 10 ml benzene) was added drop by drop. The addition process lasted approximately one hour, after which the mixture was refluxed for three hours. The progress of the reaction was monitored using thin-layer chromatography (TLC). At the end of the reaction, cold water was added, and the resulting mixture was filtered to isolate the precipitate. The precipitate was then washed with diethyl ether. The final product obtained was a gray powder with a molecular weight (Mw) of 248.7 g/mol, a melting point of 202°C, an Rf value of 0.8, and a yield of 90%.

The synthesis of 4-((2-aminothiazol-4-yl) amino) benzene-sulfonamide C_9_H_10_N_4_O_2_S_2_ (M2) 2 mmol was done by dissolving 0.53 gm of the compound in 50 ml of 99% ethanol and 2 mmol (0.17 gm) of thiourea was added. The reaction mixture was subjected to reflux for 8 hours, followed by solvent evaporation and subsequent recrystallization using ether [20]. The final product obtained was a beige powder with an Mw of 270.3g/mol, a melting point of 105°C, an Rf value of 0.75, and a yield of 86%.

The Schiff base, (Z)-4-((2-((4-hydroxybenzylidene) amino) thiazol-4-yl) amino) benzene sulfonamide was synthesized by dissolving M2 (2.05 mmol, 0.554 gm) and an equimolar amount of benzaldehyde derivatives in 50 ml absolute ethanol, with two drops of glacial acetic acid. The reaction mixture was then subjected to reflux for 24 hours. After that, the excess solvent was evaporated, and the precipitate was recrystallized from ethanol [21]. The products obtained were M3 C_16_H_14_N_4_O_3_S_2_, yellow powder, Mw=374.4 g/mol, melting point=163, Rf=0.95, % yield=90% and M4 C_18_H_19_N_5_O_2_S_2_, red powder, Mw=401.5 g/mol, melting point=167, Rf=0.79, % yield =92%.

**Figure 1:**
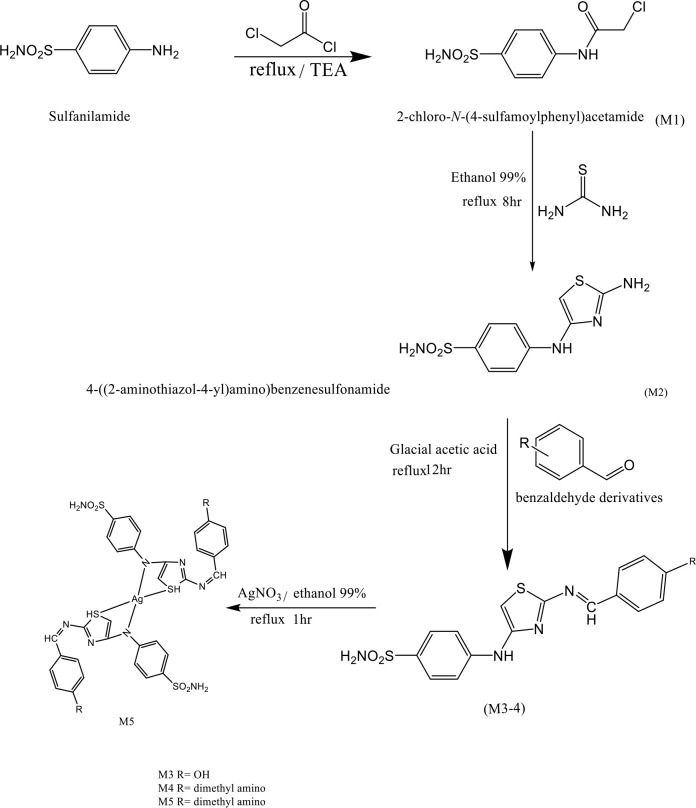
Synthesis of final compounds and intermediates

For the preparation of complex M5 (C_36_H_38_AgN_10_O_4_S_4_), silver nitrate (0,339 g, 2 mmol) was dissolved in 99% ethanol, followed by the addition of M4 (1,606 g, 4 mmol) dissolved in 99% ethanol for the production of compound F7 or F8, respectively. This solution was mixed with silver nitrate solution and refluxed for one hour. The final mixture was concentrated by drying the solvent, and the precipitate was washed with ethanol and allowed to dry [22], resulting in a brown powder with an Mw of 910.9g/mol, a melting point of 212°C, and a yield of 70% (Figure 1).

### Molecular docking study

Molecular docking studies were conducted using the Molecular Operating Environment (MOE) software, version 2015.10. The process involved preparing both the protein and ligand structures for simulation. The preparation of ligands in MOE included the protonation of 3D structures, the addition of partial charges, and energy minimization to get stable conformations. 4z0q protein was retrieved from the Protein Data Bank (PDB) (www.rcsb.org) and processed by deleting water molecules and other non-essential sites on the carbonic anhydrase IX to enable engagement with ligands. The deleted protons (to facilitate the retrieval and deposition of the protein from the PDB) were added, broken bonds were fixed, and the potential of the protein molecule was determined. MOE was utilized to identify the active site of carbonic anhydrase IX, facilitating the recognition of key amino acids within the site crucial for ligand binding. The docking simulations explored various binding modes of ligands within the receptor site of the 4z0q protein. The structural arrangements of the compounds, particularly in their deprotonated states, revealed that the negatively charged nitrogen atom in the sulfonamide group forms a coordination bond with the Zn(II) ion within the catalytic site. Additionally, this binding interaction was strengthened by an intricate network of hydrogen bonds that engaged the Thr199 residue. To evaluate the activity of the compound, we used the S score and the root mean square deviation (RMSD). The S score represents the binding affinity between the ligand entity and the target protein, with lower scores suggesting higher affinity, and the RMSD values represent the average distance between the atoms of the posed ligand and the ligand for the site of the anti-cancer that was tested.

### In-vitro cytotoxicity evaluation

To evaluate the antitumor activity of the newly synthesized sulfonamide carbonic anhydrase inhibitors (compounds M3, M4, and M5), we employed cell line assays using 3-(4,5-dimethylthiazol-2-yl)-2,5-diphenyltetrazolium (MTT). Two cell types were utilized in this study: MCF-7, a breast cancer cell line, to assess the effects of the compounds on cancer cells, and MCF10A, a normal breast epithelial cell line, to evaluate the effects on non-cancerous cells. These cell lines were procured from the Iranian National Cell Bank at the Pasteur Institute. MCF-7 cells were cultured in RPMI-1640 medium, while MCF10A cells were cultured in DMEM: F12 medium. Both media were supplemented with 10% fetal bovine serum (FBS), followed by the addition of antibiotics (100 µg/ml streptomycin and 100 units/ml penicillin). The cells were maintained in a humidified atmosphere at 37°C with 5% carbon dioxide. For cell passaging, a trypsin/EDTA solution (Gibco) and phosphate-buffered saline (PBS) were utilized. Cell viability was assessed using MTT from Sigma-Aldrich. Each type of cell (MCF-7 and MCF10A) was cultured separately in 96-well plates (1.4 × 10^4 cells per well with 200 µl of fresh medium) after a full day had passed (temperature of 37°C in a 5% CO_2_ environment). The supernatant was removed, and MTT solution at a concentration of 200 µl was added to each well and then incubated for four hours at a temperature of 37°C. After the end of the incubation period and to dissolve the formazan crystals, 100 µl of dimethyl sulfoxide (DMSO) was added, and the plate was left in a shaker at a temperature of 37°C until it was confirmed that all the crystals had completely dissolved. An ELISA reader (Model Wave xs2, BioTek) was used to measure cell vitality by absorption at 570 nm and calculate the IC50 values.

## RESULTS

### Synthesis and characterization of compounds

**Compound M1 IR** (cm^-1^): 3,327, 3,215 (N-H asymmetric and symmetric stretching vibration of primary amine), 1,689 (C=O stretching band of amide), 1,602, 1,546 (C=C stretching band of aromatic ring), 1,406 (C-N stretching band), 1,319, 1,157 (SO2 asymmetric and symmetric stretching vibration respectively), 839 (C-S stretching band), 684 (C-Cl stretching band).

**Compound M2 IR** (cm^-1^): 3,469, 3,373 (N-H asymmetric and symmetric stretching band of primary amine, respectively), 3,331, 3,242 (N-H asymmetric and symmetric stretching band of primary sulfonamide, respectively), 3,180 (N-H stretching band of secondary amine), 3,074 (C-H stretching band of aromatic ring), 1,629 (C-H bending band), 1,600 (C=N stretching band of thiazole ring), 1,510 (C=C stretching band of aromatic ring), 1,404 (C-N stretching band), 1,309, 1,151 (SO2 asymmetric and symmetric stretching vibration, respectively).

**Compound M3 IR** (cm^-1^): 3,469, 3,414 (N-H asymmetric and symmetric stretching band of primary sulfonamide, respectively), 3,244 (N-H stretching band of secondary amine), 3,500-3,200 (O-H stretching band of phenol), 1,602 (C=N stretching band of thiazole overlapping with stretching band of imine), 1514-1448 (C=C stretching band of aromatic ring), 1,400 (C-N stretching band), 1,313, 1,155 (SO_2_ asymmetric and symmetric stretching vibration, respectively).

^1^HMNR (ppm): 10.75 1H Singlet, of phenol, 9.7 1H Singlet, of Schiff base, 7.8, 7.7, 7.5, 7.4, 7.1 9H Multiplate, Signals arise due to the overlap of aromatic and heteroaromatic protons that are not equivalent, 6.9 2H Singlet, of sulfonamide group, 4.16 1H Singlet, of secondary amine.

^13^CNMR (ppm): 127-130, all of them singlet of aromatic carbon, 132 of thiazole ring, 141 of aromatic carbon linked with a secondary amine, 159 of thiazole ring, 162 of aromatic carbon linked with a hydroxyl group, 163 singlets of carbon of Schiff base, 180.99 of thiazole ring.

**Compound M4 IR** (cm^-1^): 3,415, 3,228 (asymmetric and symmetric stretching sulfonamide NH2, respectively), 3,041, 2,914 (asymmetric and symmetric stretching band of CH3, respectively), 1,585 (C=N stretching band of thiazole overlapping with stretching band of imine), 1525 (C=C stretching band of aromatic ring), 1,330 (C-N stretching band overlapping with asymmetric band of SO2), 1,330, 1,151 (SO_2_ asymmetric and symmetric stretching vibration respectively).

^1^HMNR (ppm): 9.58 (1H singlet, Schiff base), 7.8, 7.7, 7.5, 7.4, 7.1 (9H multiplate, overlapping aromatic and heteroaromatic protons), 6.9 (2H singlet, sulfonamide group), 4.1 (1H singlet, secondary amine), 3.0 (6H singlet, dimethyl amine).

^13^CNMR (ppm): 112.3-127.4 (aromatic carbon, singlets), 127.8 (thiazole ring), 132 (aromatic carbon linked with secondary amine), 152 (thiazole ring), 154 (aromatic carbon linked with dimethyl amino group), 161 (carbon of Schiff base, singlet) 184 (thiazole ring), 45.7 (carbons of methyl group).

**Compound M5 IR** (cm^-1^): 3,452, 3,228 (asymmetric and symmetric stretching sulfonamide NH2, respectively), 3,041 (asymmetric stretching band of CH3), 1608 (N-H bending vibration), 1,583 (C=N stretching band of thiazole ring), 1,544 (C=N stretching band of imine), 1,336 (C-N stretching band overlapping with asymmetric band of SO_2_), 1,336, 1,157 (SO2 asymmetric and symmetric stretching vibration respectively).

### Molecular docking findings

**Figure 2:**
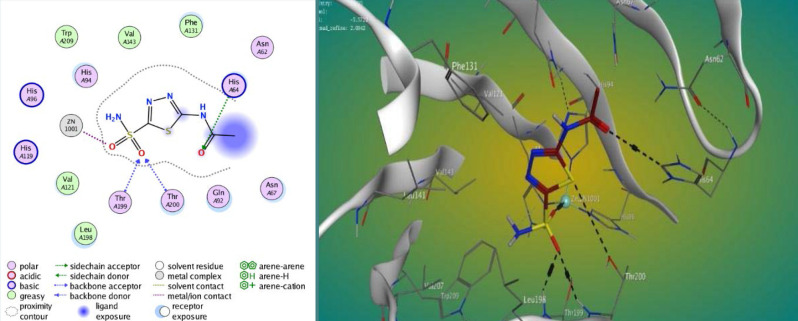
Docked pose of AZZ within the active site of CA IX (CODE: 4Z0Q)

**Figure 3:**
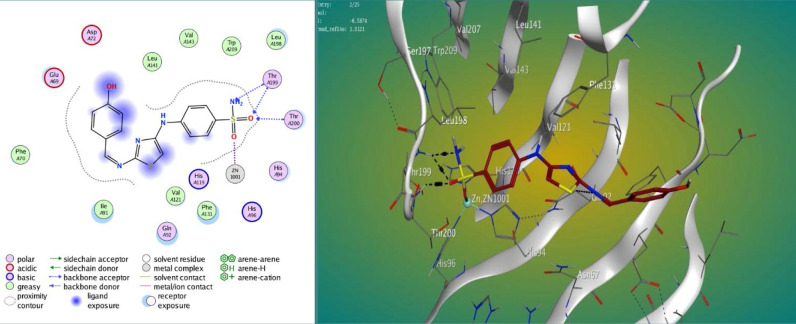
Docked pose of compound M3 within the active site of CA IX (CODE: 4Z0Q)

**Figure 4:**
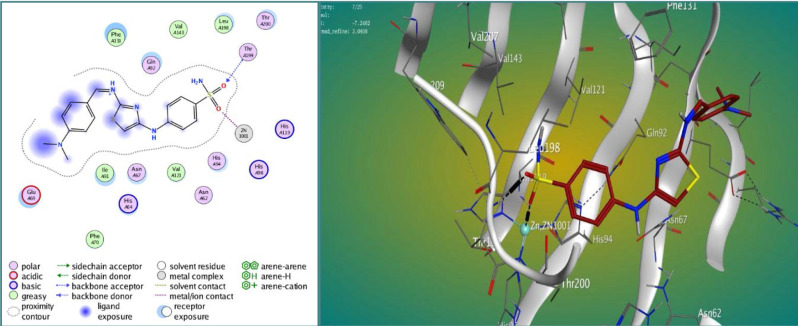
Docked pose of compound M4 within the active site of CA IX (CODE: 4Z0Q)

The findings displayed varying degrees of binding strengths among the synthesized compounds, with M3 and M4 showing strong interactions at the active site of carbonic anhydrase IX (Figures 2–4). The lower S scores and RMSD values suggest a higher affinity of these compounds for the target protein compared to the standard acetazolamide (AAZ). Table 1 shows the results of the docking study.

**Table 1:** Results of docking study of prepared compounds and standards

Compound	S scores	RMSD	Molecule engaged in binding
AZZ	-5.5722	2.0942	Zn1001, Thr A199, Thr A200, His A 64
M3	-6.587	1.312	Zn1001, Thr A199, Thr A200
M4	-7.2462	2.040	Zn1001, Thr A199

### In-vitro cytotoxicity evaluation

These synthesized compounds were compared to acetazolamide and cisplatin to benchmark their cytotoxic activities. The justification for selecting these two agents is that AAZ is the prototype for carbonic anhydrase inhibitors and has been extensively studied for its potential as an antineoplastic agent. Numerous investigations explored its activity and effectiveness in combating cancerous growth. Furthermore, carbonic anhydrase inhibitors that specifically target antineoplastic activity are currently undergoing clinical trials. These inhibitors have shown potential in selectively inhibiting carbonic anhydrase enzymes involved in cancer progression and metastasis. Cisplatin is an antineoplastic drug used for solid tumors, characterized by its inclusion of a platinum metal ion in its chemical composition. Due to its well-established antineoplastic activity, cisplatin is a valuable reference compound for studying the cytotoxic properties of synthesized compounds. The IC50 values of the prepared compounds and standards are presented in Table 2.

**Table 2:** Inhibition activity of prepared compounds and standards

Compound	MCF-7		MCF10A	
	IC50 (µM) ± SD	p-value	IC50 (µM) ± SD	P value
AZZ	67.53±1.80	Standard	53.91±5.636	Standard
Cisplatin	15.09±2.25	Standard	13.55±3.45	Standard
M3	87.65±2.16	0.0026^a^	135.18±4.19	0.0001^a^
		0.0001^b^		0.0001^b^
M4	77.43±3.31	0.0338^a^	52.51±3.31	0.894^a^
		0.0001^b^		0.0001^b^
M5	18.53±2.26	0.0001^a^	19.81±3.80	0.0001^a^
		0.271^b^		0.105^b^

a Significantly different from acetazolamide (P<0.05); ^b^Significantly different from cisplatin (P<0.05)

## DISCUSSION

The docking analysis highlights the favorable binding interactions and possible binding affinity demonstrated by the synthesized compounds, specifically M3 and M4, towards the active region of the target protein. The sulfonamide group is believed to have a crucial role in enabling these interactions. Furthermore, the presence of the thiazole ring likely contributes to the interaction of the compound with the active site of the receptor and enhances the orientation of the imine group. Compounds M3 and M4 demonstrated more robust interactions with the target protein compared to acetazolamide. Compound M4 showed the lowest S score among the studied compounds, indicating a stronger binding interaction and a potentially superior ligand-protein complex. On the other hand, compound M3 had a lower RMSD, indicating a closer alignment between the predicted and actual ligand poses. In comparison, acetazolamide demonstrated the highest S score and RMSD values. These results suggest that the synthesized compounds may have potential as novel inhibitors of carbonic anhydrase IX, which is a promising target for anticancer therapy.

Regarding MCF-7, compounds M3 and M5 show significant differences from AZZ and cisplatin, and they also demonstrated significant differences from the standards in the MCF10A cell line. In the case of the silver complex, complexation increases the activity of compound M5 (IC50=18.53 µg/ml), demonstrating a potency that is approximately four times greater than that of the parent compound M4 (IC50=77.43 µg/ml), aligning with our initial expectations. The IC50 value of M5 closely approximates that of the established standards. However, it is important to acknowledge that this inhibitory effect also extends to normal cells, and there is no significant difference from cisplatin. Our results align with previous studies suggesting that the integration of sulfonamide-bearing heterocycles increases the ability of the compound to inhibit the carbonic anhydrase enzyme [23-26].

These results suggest that the synthesized compounds may have potential as novel inhibitors of carbonic anhydrase IX, which is a promising target for anticancer therapy. However, further experimental validation is needed to confirm the efficacy and selectivity of these compounds in vivo. To address the issue of the silver complex's lack of selectivity as a drug, various solutions can be considered. Future work may focus on developing prodrugs or the conjugation of the silver complex with targeting moieties. Another approach could be investigating nanoencapsulation techniques to create a drug delivery system that targets specific cells or tissues, thereby reducing non-specific interactions with normal cells.

## CONCLUSION

This study successfully synthesized and characterized a series of thiazole-sulfanilamide derivatives, M3, M4, and M5, using sulfanilamide as a foundational precursor. Advanced spectroscopic analysis, encompassing FT-IR, ^1^H-NMR, and ^13^C-NMR techniques, confirmed the structural integrity of these compounds. Their cytotoxic activity against the human cancer cell line MCF-7 and the normal epithelial breast cell MCF10A was evaluated using the MTT assay. Compound M5 (which had a silver complex) demonstrated increased cytotoxic activity non-selectively for both cancer and normal cells, with the most pronounced effect observed on cancer cells, making it a promising compound as an antineoplastic agent.

## Personal thanks

The authors would like to thank Dr. Fahad Ghalib for all his advice, guidance, and generous assistance during the writing.

## References

[1] Sung H, Ferlay J, Siegel RL, Laversanne M, Soerjomataram I, Jemal A (2021). Global Cancer Statistics 2020: GLOBOCAN Estimates of Incidence and Mortality Worldwide for 36 Cancers in 185 Countries. CA Cancer J Clin.

[2] Naeem M, Hayat M, Qamar SA, Mehmood T, Munir A, Ahmad G (2019). Risk factors, genetic mutations and prevention of breast cancer. Int J Biosci.

[3] Angeli A, Kartsev V, Petrou A, Lichitsky B, Komogortsev A, Pinteala M (2022). Pyrazolo[4,3-c]pyridine Sulfonamides as Carbonic Anhydrase Inhibitors: Synthesis, Biological and In Silico Studies. Pharmaceuticals (Basel).

[4] Khushal A, Mumtaz A, Shadoul WA, Zaidi SHM, Rafique H, Munir A (2022). Synthesis, carbonic anhydrase II/IX/XII inhibition, DFT, and molecular docking studies of hydrazide-sulfonamide hybrids of 4-methylsalicyl-and acyl-substituted hydrazide. Biomed Res Int.

[5] Noor HN (2017). β-Carbonic Anhydrase as a Target for Eradication of Mycobacterium tuberculosis. OAJPR.

[6] McDonald PC, Chafe SC, Supuran CT, Dedhar S (2022). Cancer Therapeutic Targeting of Hypoxia Induced Carbonic Anhydrase IX: From Bench to Bedside. Cancers (Basel).

[7] Martínez-Montiel M, Romero-Hernández LL, Giovannuzzi S, Begines P, Puerta A, Ahuja-Casarín AI (2023). Conformationally Restricted Glycoconjugates Derived from Arylsulfonamides and Coumarins: New Families of Tumour-Associated Carbonic Anhydrase Inhibitors. Int J Mol Sci.

[8] Jia T, Ming SX, Cao QQ, Xu FL (2020). Combined treatment with acetazolamide and cisplatin enhances the chemosensitivity of human head and neck squamous cell carcinoma TU868 cells. Arch Oral Biol.

[9] Pustenko A, Nocentini A, Gratteri P, Bonardi A, Vozny I, Žalubovskis R (2020). The antibiotic furagin and its derivatives are isoform-selective human carbonic anhydrase inhibitors. J Enzyme Inhib Med Chem.

[10] Kalinin S, Malkova A, Sharonova T, Sharoyko V, Bunev A, Supuran CT (2021). Carbonic Anhydrase IX Inhibitors as Candidates for Combination Therapy of Solid Tumors. Int J Mol Sci.

[11] Güttler A, Theuerkorn K, Riemann A, Wichmann H, Kessler J, Thews O (2019). Cellular and radiobiological effects of carbonic anhydrase IX in human breast cancer cells. Oncol Rep.

[12] Khushal A, Mumtaz A, Shadoul WA, Zaidi SHM, Rafique H, Munir A, Deniz NG (2022). Synthesis, Carbonic Anhydrase II/IX/XII Inhibition, DFT, and Molecular Docking Studies of Hydrazide-Sulfonamide Hybrids of 4-Methylsalicyl- and Acyl-Substituted Hydrazide. BioMed Res Int.

[13] Bonardi A, Nocentini A, Bua S, Combs J, Lomelino C, Andring J (2020). Sulfonamide Inhibitors of Human Carbonic Anhydrases Designed through a Three-Tails Approach: Improving Ligand/Isoform Matching and Selectivity of Action. J Med Chem.

[14] Čapkauskaitė E, Zubrienė A, Paketurytė V, Timm DD, Tumkevičius S, Matulis D (2018). Thiazole-substituted benzenesulfonamides as inhibitors of 12 human carbonic anhydrases. Bioorg Chem.

[15] Arshad MF, Alam A, Alshammari AA, Alhazza MB, Alzimam IM, Alam MA (2022). Thiazole: A Versatile Standalone Moiety Contributing to the Development of Various Drugs and Biologically Active Agents. Molecules.

[16] Petrou A, Fesatidou M, Geronikaki A (2021). Thiazole Ring-A Biologically Active Scaffold. Molecules.

[17] Supuran CT, Scozzafava A, Casini A (2003). Carbonic anhydrase inhibitors. Med Res Rev.

[18] Naji EM, Hussein SA, Naser NH (2023). Evaluation of newly synthesized compounds targeting carbonic anhydrase enzyme for antineoplastic activity in solid tumors. J Contemp Med Sci.

[19] Jawad HA, Naser NH, Alwash AH, Hussein SA (2023). Design, synthesis, in Silico study and preliminary pharmacological assessment of new ciprofloxacin analogues having thiazole nucleus. J Pharm Negat Results.

[20] Doregiraee A, Tavakolinejad Kermani E, Khabazzadeh H, Pouramiri B (2015). Synthesis of new 1,3-thiazole derivatives; Using 1-(4-carbamoylphenyl)-3-methylthiourea and 1-methyl-3-(quinolin-8-yl) thiourea as starting materials. J Chil Chem Soc.

[21] Sunjuk M, Al-Najjar L, Shtaiwi M, El-Eswed B, Al-Noaimi M, Al-Essa L (2022). Transition Metal Complexes of Schiff Base Ligands Prepared from Reaction of Aminobenzothiazole with Benzaldehydes. Inorganics.

[22] Pavia DL, Lampman GM, Kriz GS, Vyvyan JA (2001). Introduction to Spectroscopy.

[23] Kılıcaslan S, Arslan M, Ruya Z, Bilen Ç Ergün A, Gençer N (2016). Synthesis and evaluation of sulfonamide-bearing thiazole as carbonic anhydrase isoforms hCA I and hCA II. J Enzyme Inhib Med Chem.

[24] Abas M, Bahadur A, Ashraf Z, Iqbal S, Rajoka MSR, Rashid SG (2021). Designing novel anticancer sulfonamide based 2,5-disubstituted-1,3,4-thiadiazole derivatives as potential carbonic anhydrase inhibitor. J Mol Struct.

[25] Manzoor S, Angeli A, Zara S, Carradori S, Rahman MA, Raza MK (2022). Development of benzene and benzothiazole-sulfonamide analogues as selective inhibitors of the tumor-associated carbonic anhydrase IX. Eur J Med Chem.

[26] Buza A, Türkeş C, Arslan M, Demir Y, Dincer B, Nixha AR (2023). Discovery of novel benzenesulfonamides incorporating 1,2,3-triazole scaffold as carbonic anhydrase I, II, IX, and XII inhibitors. Int J Biol Macromol.

